# Life-History Traits of *Macrolophus pygmaeus* with Different Prey Foods

**DOI:** 10.1371/journal.pone.0166610

**Published:** 2016-11-21

**Authors:** Serigne Sylla, Thierry Brévault, Karamoko Diarra, Philippe Bearez, Nicolas Desneux

**Affiliations:** 1 Université Cheikh Anta Diop (UCAD), Equipe Production et Protection Intégrées en Agroécosystèmes Horticoles - 2PIA, Faculté des Sciences et Techniques, Dakar, Senegal; 2 BIOPASS, ISRA-UCAD-IRD, Dakar, Senegal; 3 CIRAD, UPR AIDA, F-34398 Montpellier, France; 4 INRA (French National Institute for Agricultural Research), Univ. Nice Sophia Antipolis, CNRS, UMR 1355–7254, Institut Sophia Agrobiotech, 06903, Sophia Antipolis, France; Universita degli Studi della Basilicata, ITALY

## Abstract

*Macrolophus pygmaeus* Rambur (Hemiptera: Miridae) is a generalist predatory mirid widely used in augmentative biological control of various insect pests in greenhouse tomato production in Europe, including the invasive tomato leafminer, *Tuta absoluta* (Meyrick) (Lepidoptera, Gelechiidae). However, its biocontrol efficacy often relies on the presence of alternative prey. The present study aimed at evaluating the effect of various prey foods (*Ephestia kuehniella* eggs, *Bemisia tabaci* nymphs, *Tuta absoluta* eggs and *Macrosiphum euphorbiae* nymphs) on some life history traits of *M*. *pygmaeus*. Both nymphal development and adult fertility of *M*. *pygmaeus* were significantly affected by prey food type, but not survival. Duration of nymphal stage was higher when *M*. *pygmaeus* fed on *T*. *absoluta* eggs compared to the other prey. Mean fertility of *M*. *pygmaeus* females was greatest when fed with *B*. *tabaci* nymphs, and was greater when offered *M*. *euphorbiae* aphids and *E*. *kuehniella* eggs than when offered *T*. *absoluta* eggs. Given the low quality of *T*. *absoluta* eggs, the efficacy of *M*. *pygmaeus* to control *T*. *absoluta* may be limited in the absence of other food sources. Experiments for assessing effectiveness of generalist predators should involve the possible impact of prey preference as well as a possible prey switching.

## Introduction

The tomato leaf miner, *Tuta absoluta* (Meyrik) (Lepidoptera, Gelechiidae) is a major invasive pest. Originating from South America, *T*. *absoluta* was first detected in Spain in 2006 and has spread to several European, Middle Eastern, Africa North of the Sahel and sub-Saharan Africa countries [1–3]; the infestation is likely to persist even in Northern parts of the Eurasian continent [2] as the pest is able to overwinter successfully e.g. in Belgium [4]. Losses can reach 100% of both field and greenhouse production for fresh market due to leaf mining and fruit damage. Tomato growers often rely on systematic use of insecticides to control *T*. *absoluta* infestations, with potentially undesired side effects on non-target organisms [5,6], and potential selection of insecticide-resistant *T*. *absoluta* populations [7,8]. Integrated pest management (IPM) is promoted by FAO and Europe (Directive 2009/128/EC) as a sustainable approach to crop protection that minimizes the use of pesticides. It is based on the combination of preventive methods and monitoring of pests and their damage, but also on the use of biological, physical, and other sustainable non-chemical methods if they provide suitable pest control. Biological control (BC) which relies on the use of living organisms (natural enemies) to reduce pest populations is a key component of IPM [1,9,10]. It includes classical (introduction of natural enemies to a new area), augmentation (supplemental release of natural enemies), and conservation BC (habitat managed to favor natural enemies). However, biological control is not widely implemented in pest management programs, mostly due to growers’ lack of knowledge on biology and ecology of both pests and their natural enemies.

Generalist predators are known to greatly contribute to biological control of many agricultural pests in the word [11]. In the last five years, studies have documented the biology and effectiveness of the zoophytophagous predatory *Macrolophus pygmaeus* Rambur (Hemiptera, Miridae) to control various crop pests [12,13] Those predatory mirids are efficient natural enemies for controlling whiteflies, thrips, aphids, mites and lepidopteran pests [14–17]. Recent results showed that *M*. *pygmaeus* is also a suitable predator of the invasive pest *T*. *absoluta* [2,10,18,19], This predatory mirid is a key component of newly developed integrated pest management (IPM) for tomato crops in Europe. However, predatory mirids need alternative prey to establish and increase their populations [20]. For example, studies showed that *M*. *pygmaeus* populations increase when they feed on *Ephestia kuehniella* (Lepidoptera, Pyralidae) eggs and *Artemia* cysts as alternative food sources [21–23]. Moreover, it has been shown that *T*. *absoluta* on tomato plants as exclusive food source was insufficient to obtain a significant and stable *M*. *pygmaeus* population, compared to feeding on *E*. *kuehniella* eggs on tomato [20]. However, the association of *Bemisia tabaci* (Gennadius) (Hemiptera, Aleyrodidae) and *T*. *absoluta* as food source for *M*. *pygmaeus* provides effective pest control [24,25]. *Macrosiphum euphorbiae* (Thomas) and *Myzus persicae* (Sulzer) (Homoptera, Aphididae) are the rare aphid species that can survive on tomato plants [26]. Some studies indicate that *Macrolophus basicornis* (Hemiptera: Miridae) can survive and reproduce with *M*. *euphorbiae* aphids as prey, but that this food source negatively affects female fertility [27]. Studies on the seasonal abundance of aphids and their natural enemies in tomato fields in 1992–1993 in Greece showed that *M*. *pygmaeus* was the most important predator of aphids [26,28]. *M*. *pygmaeus* develops also well on the aphid *M*. *persicae* on pepper and tomato [26,29]. However, little is known on *M*. *pygmaeus* fitness when feeding of *M*. *euphorbiae*. The present study aimed at comparing nymphal development time and reproductive performance of *M*. *pygmaeus* when preying *T*. *absoluta* eggs, *E*. *kuechniella* eggs, *B*. *tabaci* nymphs, or *M*. *euphorbiae* aphids.

## Materials and Methods

### Plants and insects

Plants used in the experiments were 5 week-old tomato plants, *Solanum lycopersicum* L. (cv Marmande) grown in climatic chambers at 24 ± 1°C, 60 ± 5% RH, and photoperiod16L: 8D. *T*. *absoluta*, *B*. *tabaci* and *M*. *euphorbiae* were reared on caged tomato plants (120 x 70 x 125 cm) in climatic chambers at 24 ± 1°C, 60 ± 5% RH, and photoperiod16L: 8D. Both *B*. *tabaci* and *T*. *absoluta* insects originated from a lab colony, respectively reared on tobacco and tomato plants. *M*. *euphorbiae* aphids were collected from INRA-ISA tomato greenhouses. *M*. *pygmaeus* adults and *E*. *kuehniella* eggs were provided by Biotop (Livron-sur-Drôme, France).

### Feeding bioassays

Development time and juvenile survival of *M*. *pygmaeus* were assessed according to different food sources: (a) *T*. *absoluta* eggs, (b) *B*. *tabaci* nymphs, (c) *M*. *Euphorbiae* nymphs and (d) *E*. *kuehniella* eggs. Newly emerged *M*. *pygmaeus* nymphs (at stage N1) were individually transferred into 10-ml tubes with one tomato leaflet. Every two days, tubes were checked for nymphal stage. Food was supplied every two days and the quantity offered depended on the nymphal stage of the predator. Food quantity offered to each nymphal stage was estimated following a preliminary experiment in the laboratory. *M*. *pygmaeus* nymphal stages N1, N2, N3, N4, and N5, were respectively offered 10, 18, 24, 32, 36 *T*. *absoluta* eggs, 8, 12, 16, 24, 24, 28 *E*. *kuehniella* eggs, 20, 24, 24, 40, 40 *B*. *tabaci* nymphs, and 20, 20, 30, 30, 30 *M*. *euphorbiae* nymphs. The tomato leaflet was changed when necessary. Nymphal development and survival were checked daily until either death or adulthood. Nymphs that died on the first day of the experiment were replaced by new ones, as it was assumed that this was not due to prey food. Each test was replicated 30 times.

Ten newly emerged pairs of *M*. *pygmaeus* adults originating from the previous bioassay were transferred to ventilated plastic cups (7 cm-diameter, 10 cm-height) containing 5-week old tomato plants. *M*. *pygmaeus* adults were fed with respective food until the female died. Each pair was transferred to a new plastic cup with another tomato plant every 4 days. For each plastic cup, total offspring (first-instar nymphs) produced per female was recorded twelve days later because, by counting nymphs, as eggs laid by *M*. *pygmaeus* on plant stems are hardly visible.

### Statistical analyses

Analyses were performed with the R software version 3.2.2 (R Development Core Team). Prior to analysis, data from experiment were tested for normality (Shapiro-Wilk test) and homogeneity of variances (Bartlett test). Development time (from N1 to N5) of nymphs and fecundity (number of first instar nymphs produced per female) were analyzed using generalized linear models (GLM) based respectively on a Poisson (link = log) and a Gaussian (link = identity) distribution. Post hoc multiple comparisons of mean values were performed using the Newman–Keuls method (package *multcomp*). Survival rates were compared using a Kaplan Meier survivorship test (SPSS).

## Results

**Fig 1:**
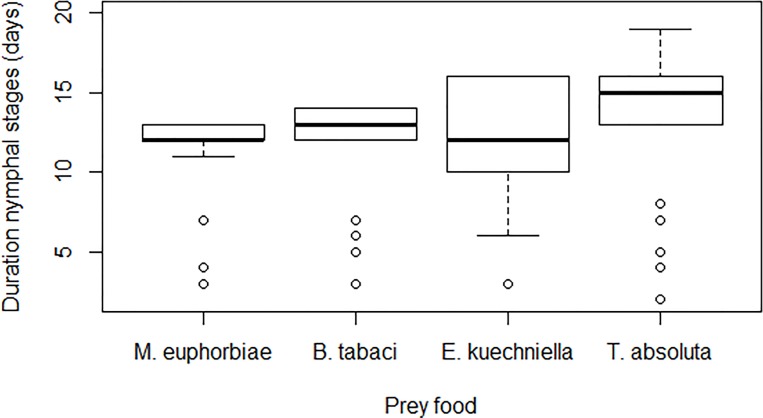
Median duration of nymphal stages (days ± SEM) of *Microlophus pygmaeus* fed on *Tuta absoluta* eggs, *Ephestia kuehniella* eggs, *M*. *euphorbiae* nymphs or *Bemisia tabaci* nymphs. Bars topped by same letter are not statistically different (P < 0.05).

**Fig 2:**
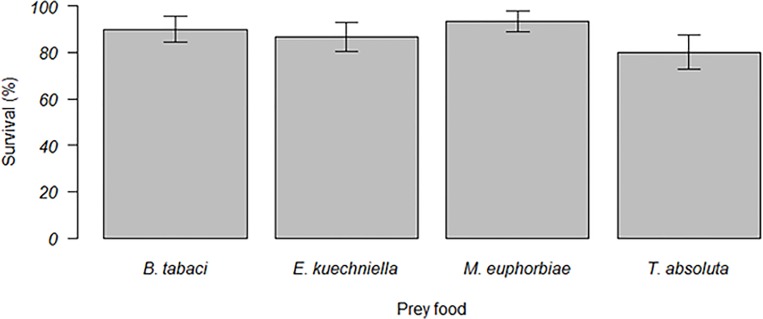
Mean survival (± SEM) of immature stages of *Macrolophus pygmaeus* fed on *Tuta absoluta* eggs, *Ephestia kuehniella* eggs, *Macrosiphon euphorbiae* nymphs or *Bemisia tabaci* nymphs.

**Fig 3:**
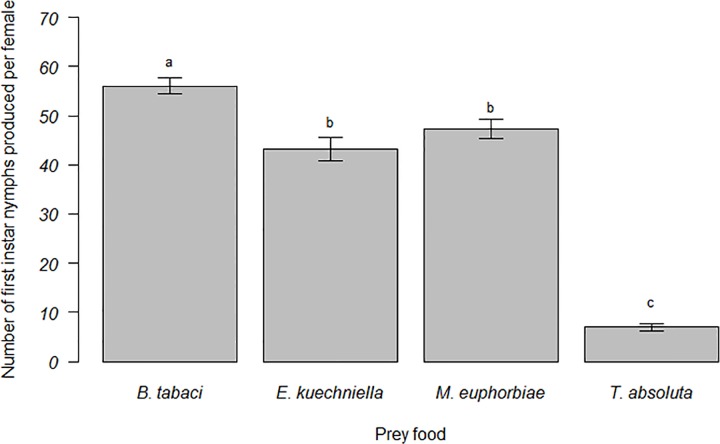
Mean fertility (number of first-instar nymphs ± SEM) of *Macrolophus pygmaeus* fed on *Tuta absoluta* eggs, *Ephestia kuehniella* eggs, *Macrosiphon euphorbiae* nymphs or *Bemisia tabaci* nymphs. Bars topped by same letter are not statistically different (P < 0.05).

A significant effect of prey food on the development time (N1 to N5) of *M*. *pygmaeus* was observed (F_3, 103_ = 16.6, *P* < 0.001). *M*. *pygmaeus* required more time to reach the adult stage when offered exclusively *T*. *absoluta* eggs, compared to *E*. *kuehniella* eggs, *M*. *euphorbiae* and *B*. *tabaci* nymphs (Fig 1). However, prey food did not affect survival of *M*. *pygmaeus* Kaplan Meier survivorship (Breslow Generalized Wilcoxon test); χ^2^ = 3.182; df = 3; *P* = 0.364 (Fig 2). A significant effect of prey food on the number of first-instar nymphs produced per female was observed (F_3, 36_ = 142.9, *P* ˂ 0.001). Mean fertility of *M*. *pygmaeus* females was greatest when fed with *B*. *tabaci* nymphs, and was greater when offered *M*. *euphorbiae* aphids and *E*. *kuehniella* eggs than when offered *T*. *absoluta* eggs (Fig 3).

## Discussion

The present study showed a longer duration of nymphal development and lower fertility of *M*. *pygmaeus* when fed with *T*. *absoluta* eggs, compared to other prey foods such as *E*. *kuehniella* eggs, *B*. *tabaci* nymphs and *M*. *euphorbiae* nymphs. Our results support a previous study showing that fertility was lower when *M*. *pygmaeus* were fed with *T*. *absoluta* eggs compared to *E*. *kuehniella* eggs [20]. However, authors did not show significant differences between prey foods regarding development time. *T*. *absoluta* eggs are probably of low nutritional quality for the generalist predator *M*. *pygmaeus*, and its role as a biocontrol agent is probably limited in the absence of other food sources. Other studies showed that *M*. *pygmaeus* can exhibit prey switching when foraging in patches with disproportionate densities of *T*. *absoluta* and *B*. *tabaci* [30]. This particular behavior might result in effective regulation of both prey populations [24,25]. The same phenomenon has been observed for the generalist predator, *Orius insidiosus* (Hemiptera:Anthocoridae), in presence of the soybean aphid [31,32]. Thus, alternative prey could provide good control of *T*. *absoluta* by increasing density of *M*. *pygmaeus* populations [25].

Higher fitness was observed when *M*. *pygmaeus* fed on *M*. *euphorbiae* nymphs. Our results corroborate previous studies [17,27,33–34] indicating that aphids in general are good prey for *M*. *pygmaeus*. These authors showed that *M*. *persicae* as a food source increases *M*. *pygmaeus* longevity and reproduction rate, especially when these aphids were reared on pepper plants. Thus, nutritional value of aphids is probably linked to host plant quality or aphid adaptation. Lykouressis et al. [35] reported similar trend when *Aphis fabae solanella* (Hemiptera, *Aphididae*) were fed on *Solanum nigrum* L. compared to *Dittrichia viscosa* (L.) Greuter, (*Asteraceae*). Opposite effect was observed with other aphid species. For example, development of *M*. *pygmaeus* was inhibited when fed on *A*. *gossypii* on cucumber or *Capitophorus inulae* (Homoptera: *Aphididae*) on *D*. *viscosa* [26]. Fitness of predators such as *M*. *pygmaeus* might depend not only on the type of prey food but also on the host plant of the prey. It could also depend on both the host plant and genotype of the prey. For example, fitness of *A*. *gossypii* on different host plants such as cucumber, cotton, okra and eggplant, depends on genotype (host races) [36].

Integrated pest management (IPM) strategies are being increasingly used in open field and greenhouse crops [37–39]. In the last three decades, invasive pests such as the leafminer, *Liriomyza trifolii* (Diptera: Agromyzidae), thrips, *Frankliniella occidentalis* (Thysanoptera: Thripidae) and the whitefly *B*. *tabaci* [24,25,40] have posed a major threat for the continuous production of vegetable crops. Nowadays, these pests are fully integrated in agro-ecosystems and are successfully controlled by IPM programs based on the use of natural enemies, particularly generalist predators [10]. The same trend has been experienced for the control of aphids [41,42] and *T*. *absoluta* [10,20,43]. Our results show that *M*. *euphorbiae*, as an aphid species capable of colonizing tomato crops, is of good quality as food source for *M*. *pygmaeus*. They also confirm that *B*. *tabaci* and *E*. *kuehniella* are of good quality as food source for *M*. *pygmaeus*. They could be useful for IPM programs to control *T*. *absoluta* pest when present simultaneously in tomato crops. These results indicate that experiments on predation should involve preference and prey switching of *M*. *pygmaeus* in order to assess the effectiveness of generalist predators to efficiently control *T*. *absoluta* infestations.
